# RETRACTED ARTICLE: Whole-genome assembly of *Akkermansia muciniphila* sequenced directly from human stool

**DOI:** 10.1186/s13062-015-0041-1

**Published:** 2015-02-19

**Authors:** Aurélia Caputo, Grégory Dubourg, Olivier Croce, Sushim Gupta, Catherine Robert, Laurent Papazian, Jean-Marc Rolain, Didier Raoult

**Affiliations:** 1 URMITE, UMR CNRS 7278-IRD, Aix-Marseille Université, Marseille, Cedex 5 France; 2 AP-HM, CHU Timone, Pôle Infectieux, 13005 Marseille, France; 3 Service de Réanimation Médicale-Détresse Respiratoires et Infections Sévères, Marseille, France

The Editor-in-Chief has retracted this article because the authors did not obtain informed consent to publish identifying information about the patient who provided the sample used in this research. The content of this article is no longer available online to protect patient confidentiality.

Grégory Dubourg and Sushim Gupta disagree with this retraction. The remaining authors did not respond to correspondence from the publisher about this retraction.

